# Differences in Allometric Relationship of Two Dominant Woody Species Among Various Terrains in a Desert Region of Central Asia

**DOI:** 10.3389/fpls.2021.754887

**Published:** 2021-11-10

**Authors:** Xue Wu, Xin-Jun Zheng, Xiao-Han Mu, Yan Li

**Affiliations:** ^1^College of Resources and Environment Science, Xinjiang University, Urumqi, China; ^2^Key Laboratory of Oasis Ecology, Ministry of Education, Xinjiang University, Urumqi, China; ^3^Ecological Postdoctoral Research Station, Xinjiang University, Urumqi, China; ^4^State Key Laboratory of Desert and Oasis Ecology, Xinjiang Institute of Ecology and Geography, Chinese Academy of Sciences, Urumqi, China; ^5^Fukang Station of Desert Ecology, Chinese Academy of Sciences, Urumqi, China; ^6^State Key Laboratory of Subtropical Silviculture, Zhejiang A&F University, Hangzhou, China

**Keywords:** plant height, canopy radius, basal diameter, allometric scaling, terrain

## Abstract

The allometric relationship among different functional traits is an ecological strategy for plants to promote resource utilization, which indicates the ability of plants to adapt to environmental changes coordinately. In this study, we conducted a field survey on *Haloxylon ammodendron* and *H. persicum* among different terrains (dune crest, eastern slope, western slope and inter-dune) in the Gurbantunggut Desert, obtained their quantitative and morphological characteristics, and analyzed their allometric relationships between plant height and canopy radius, plant height and basal diameter by using standardized major axis estimation. We found that: (1) The dominated terrains of *H. ammodendron* and *H. persicum* were different; (2) The individual morphology of the two *Haloxylon* species changed significantly with the terrains (*p* < 0.05), with the largest and smallest ones growing on the eastern slope and the inter-dune lowland, respectively; (3) Fixed allometric patterns were observed in the above-ground parts of the two *Haloxylon* species, as the growth of canopy and basal stem was preferentially to plant height; (4) These allometric relationships were significantly affected by the terrain, and exhibited discrepancy between two species, they both invested less in plant height in windy habitats, such as the dune crest and western slope, but *H. ammodendron* growing on the western slope and *H. persicum* growing on the eastern slope invested more in basal diameter for strengthening mechanical support and resources acquisition, respectively. These results indicated that both studied species adopted an ecological strategy that allocating more resources to horizontal expansion rather than vertical growth, the terrain has an important influence on the allometric relationship of their above-ground parts, and the trade-off mechanism of main components investing was different for these two species due to habitat heterogeneity and ecological adaptability.

## Introduction

The resources such as nutrients and water as well as carbohydrates obtained by plants through photosynthesis and root absorption determine their life processes such as survival, growth, and reproduction (Allen et al., 2008; Chen et al., 2013; Jennifer et al., 2019). However, since the resources that plants could obtain are limited, the resource distribution among various physiological/ecological functions often appears conflicting (Ogden, 1974; Liu and Ma, 2015). These conflicting resource requirements determine that plants need to balance among different functional traits, and allometric scaling is one of the most direct manifestations of such balance (Weiner, 2004). For example, with changing environment, plant biomass distributes differently in different vegetative organs (e.g., roots, stems, and leaves) or reproductive organs (e.g., flowers, fruits, and seeds), and varies between above- and below-ground parts (Cheng et al., 2014; Zhang and Peng, 2018; Chen et al., 2020; Sun et al., 2021; Zheng et al., 2021). These changes directly reflect the ecological strategies that plants adopt to cope with environmental selection pressures (Smith et al., 1985; Primack, 1987; Midgley and Bond, 1989). Allometric scaling has been proven to be an valid theory for describing the proportional relationship among plant morphological characteristics since it was first proposed, and it has been widely used because it can reveal the relationship between the size and physiological attributes of plant individuals (West et al., 1997; Lu et al., 2007; Han and Fang, 2008).

Morphological parameters such as plant height, basal diameter, and canopy radius are the most easily observed and measurable indicators in plants (Noriyuki, 2011). They partially determine the contact area of plants to external resources, and are the basis for characterizing plant growth and development (Archibald and Bond, 2003; Li S. et al., 2011). The allometric relationships among the above-ground parts of plants, that is, the relative relationship between plant height and canopy radius and that between plant height and basal diameter, has an important influence on external morphogenesis and metabolic activities of plant individuals (Zhang et al., 2009; Hou et al., 2014; Shi et al., 2015). However, the knowledge of how this allometric relationship changes along the environmental gradient is scarce, which will restrict the accurate simulation and prediction about plant growth dynamics under the background of global climate change (Lines et al., 2012).

*Haloxylon ammodendron* and *H. persicum* are typical xerophytic species with well-developed root systems, degraded leaves, and used assimilating branches to perform photosynthesis (Pyankov et al., 1999). They can resist a variety of stresses, such as barrenness, drought, salt, alkali, wind, and sand, are excellent species for wind prevention, sand fixation, and microclimate improvement in arid region (Wang et al., 2011). Within Central Asian desert ecosystems, they naturally occur and are the dominant species that widely distribute in China, Kazakhstan, Uzbekistan, Turkmenistan, and many other Central Asian countries (Buras et al., 2012). Determining the mechanisms underlying the spatial distribution of plant species is one of the central themes in biogeography and ecology (Xu et al., 2014), and the influence of terrain is one of the important research topics of this theme. The micro-habitats resulting from different terrains show strong heterogeneity in temperature, wind, soil physical and chemical properties, and directly or indirectly affecting the growth, development, and reproduction of plants, especially in the seed germination and seedling settlement stages (Li et al., 2005, Li H. et al., 2011; Xu et al., 2014; Dang et al., 2015). Exploring the effect of terrain on the two *Haloxylon* species’ distribution is conducive to provide better protection and management for them, and is also beneficial to sustain their role in maintaining local ecological safety. Studies have been done on their biomass allocating pattern between above- and below- ground parts, between stems and assimilated branches, and the allometric relationship between the area of assimilated branches and stems (Buras et al., 2012; Li Z. B. et al., 2013). However, the allometric relationship among basic functional traits such as plant height, basal diameter, and canopy radius of these two species, as well as the determinants that influencing their resource allocation in different terrains have not yet been clarified. Studying the allometric scaling of important plant functional traits and their determining factors under different terrain conditions will help us to further understand the ecological adaptation mechanisms of plants to environmental changes. For this purpose, we selected *H. ammodendron* and *H. persicum* in the southern Guerbantonggut Desert of Central Asia, Xinjiang, as the research material to explore the differences in their distribution, morphology, and the allometric scaling (plant height vs. canopy radius and plant height vs. basal diameter) under different terrain conditions. Our objective is to reveal the responses of desert plants’ distribution, structural function traits and allometry to different terrains, and deepen the understanding of desert plants adapting to varying environment.

## Materials and Methods

### Overview of the Study Area

**FIGURE 1:**
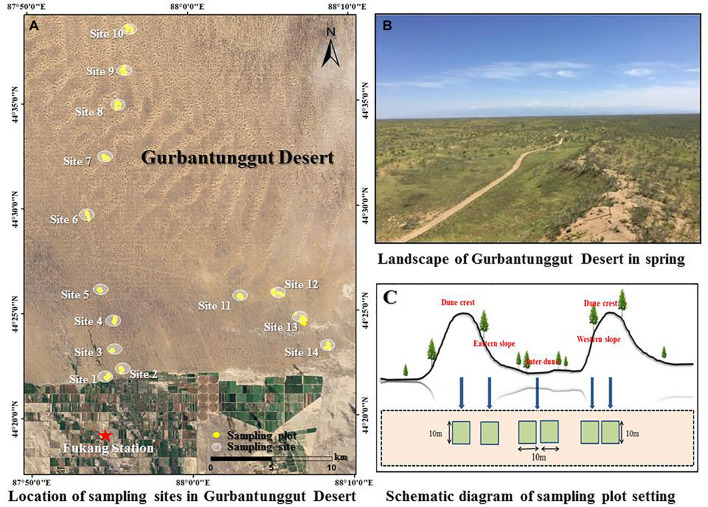
Landscape **(A,B)** and sampling diagram **(A,C)** of the study area.

The study was carried out at the vicinity of Fukang Station of Desert Ecology, Chinese Academy of Sciences (44°17′N, 87°56′E and elevation 475 m, represented by a red star in Figure 1A). The study area is located in the Gurbantunggut Desert, at the arid center of the Europe-Asia Continent. It has a typical temperate continental arid climate, with cold winters, hot summers, and large temperature differences between day and night. The annual average temperature is 6.6°C, the annual potential evapotranspiration is around 1,000 mm, and the annual precipitation is 100–150 mm. The days with snow cover are 100–160, and the maximum depth of snow cover is above 20 cm. The soil type can be divided into denser gray desert soil, salinized gray desert soil, and aeolian sandy soil with higher sand content according to the texture. The majority of the desert is covered by sand dunes and inter-dune lowland. The dunes are mainly longitudinal sand dunes, with a length up to more than 10 km, at north-south direction, and a height ranging from 10 to 50 m. There is a 10–40 m wide flowing zone on the dune crest, with slightly asymmetrical dune profiles (Wang et al., 2003). *H. ammodendron* and *H. persicum* are the dominate species in this area (Figure 1B). They are perennial shrubs or small trees belonging to the *Chenopodiaceae* family and are the most widely distributed species in the deserts of Central Asia.

### Data Acquisition

The field survey started from August 2015, and there was no rainfall 8 days before the survey started. In order to collect more comprehensive data and provide reliable information on the distribution and morphological characteristics of *H. ammodendron* and *H. persicum*, we totally sampled 14 representative sites with well-developed natural vegetation, complete community structure, and less human disturbance. These sampling sites located from the southern edge of Gurbantunggut Desert to interior at the intervals of 3–5 km (Figure 1A and Table 1). For each sampling site, adjacent dunes with similar heights were selected, and four sample plots (10 m × 10 m) were set up on the two dune crests, eastern slope, and western slope, respectively (Figure 1C). In addition, eight plots (10 m × 10 m) were set up in the inner-dune lowland due to its larger area (Figure 1C). Finally, a total of 336 plots were sampled.

**TABLE 1:** Coordinates, elevation, and stand density of 14 sampling sites.

Sampling site	Longitude (N)	Latitude (E)	Elevation (m)	Stand density (No./100 m^2^)
Site 1	44°21′51.4″	87°54′42.5″	456	6.58 ± 0.84
Site 2	44°22′17.1″	87°55′36.8″	456	17.13 ± 2.46
Site 3	44°23′12.3″	87°54′42.5″	446	22.42 ± 2.27
Site 4	44°24′36″	87°55′9.8″	453	9.17 ± 1.73
Site 5	44°26′5.7″	87°55′17.8″	455	6.92 ± 1.46
Site 6	44°29′41″	87°53′39.1″	440	1.04 ± 0.29
Site 7	44°32′60″	87°54′43.3″	449	2.88 ± 0.53
Site 8	44°35′0.3″	87°55′39″	444	1.75 ± 0.40
Site 9	44°36′35.9″	87°55′56.1″	468	2.38 ± 0.59
Site 10	44°38′25.9″	87°56′13.4″	480	0.88 ± 0.25
Site 11	44°25′49″	88°2′59.1″	467	3.92 ± 1.68
Site 12	44°25′59.4″	88°5′9.4″	458	6.67 ± 2.25
Site 13	44°24′26.4″	88°6′52.5″	456	2.75 ± 0.42
Site 14	44°23′14.3″	88°8′25.4″	469	3.83 ± 0.69

After removing the litter from the soil surface in each sampling plot, the five-point sampling method was used to collect a mixed soil sample from top to bottom (0–20 cm layer). The soil samples were placed in tin boxes and valve bags, then brought back to the laboratory. At the same time, using a cutting ring, a sample of the undisturbed soil was collected from the center of the sampling plot to determine soil bulk density.

Previous studies have shown that it is reasonable to model the shape of a desert shrub as a semi-triaxial ellipsoid with an elliptically shaped canopy (Li S. et al., 2011; Xu et al., 2017). The largest secant segment of the vertical projection of the individual on the ground surface is taken as the long axis of the canopy (a), the secant segment passing through the center perpendicular to the long axis is taken as the short axis (b), the distance from the highest point of the center to the ground is taken as the height (H). The diameter of the plant trunk on the ground is taken as the basal diameter (D) (Li S. et al., 2011). The height (H), basal diameter (D), long axis (a), and short axis (b) of each *H. ammodendron* or *H. persicum* in the plot were measured individually with a ruler. According to their basal diameter, plant individuals were divide into three development stages, seedling (D < 1.2 cm), young tree (1.2 cm ≤ D ≤ 6.5 cm), big tree (D > 6.5 cm), respectively (Jia and Lu, 2005).

### Determination of Canopy Radius

Assuming that the canopy of *H. ammodendron* or *H. persicum* has an elliptical shape, the canopy area (CA) and canopy radius (R) were calculated according to the following equation:


(1) CA=π⁢a⁢b/4

(2) $$R=\sqrt{a\,b}/2$$

where a and b are the long and short axis of the canopy, respectively.

### Determination of Soil Physical and Chemical Properties

The collected soil samples were air-dried and sieved (2 mm) to analyze physical and chemical properties, including bulk density (g/cm^3^), water content (%), pH, electrical conductivity (us/cm), organic carbon content (g/kg), total nitrogen content (g/kg), and total phosphorus content (g/kg). Soil bulk density was determined by the ring knife method. Soil water content was determined by the drying method, and on a mass basis, was converted to soil volumetric water content using the soil bulk density. Soil pH was determined by the potentiometric method (soil: water = 1:5). Soil electrical conductivity was determined by the electrical conductivity method (soil: water = 1:5). Soil organic carbon content was determined by the potassium dichromate external heating method. Soil total nitrogen content was determined by the Kelvin digestion method. Soil total phosphorus content was determined by the hydrochloric acid-hydrogen fluoride molybdenum antimony colorimetric method.

### Data Analysis

The relationship between plant height and basal diameter and between plant height and canopy radius of *H. ammodendron* or *H. persicum* in different terrains was determined using a linear regression model [*log*_10_⁡(*y*) = *log*_10_⁡(*b*) + *alog*_10_⁡(*x*)] after linearization by logarithmic transformation. *x* and *y* represent the different attribute values of *H. ammodendron* and *H. persicum*. *a* is the slope, log_10_(*b*) is the intercept, and the value of *a* (Allometric index) indicates whether the relationship between the two attributes is isometric (*a* = 1) or allometric (*a ≠* 1). A value of *a* > 1 indicates that the increase of *y* is greater than that of *x*, and *a* < 1 indicates that the increase of *y* is smaller than that of *x*. The log_10_(*b*) value does not affect the relationship. The allometric relationship between plant height (H), basal diameter (D), and canopy radius (R) were determined according to the following model (Li Y. et al., 2013):


(3) $$\log_{10}\left(D\right)=\log_{10}\left(b\right)+a\,\log_{10}\left(H\right)$$

(4) $$\log_{10}\left(R\right)=\log_{10}\left(b\right)+a\,\log_{10}\left(H\right)$$

The standardized major axis estimation was used to calculate the coefficient *a* and log_10_(*b*) of the regression equation. The principle of linear regression is to minimize the distance between the measured value and the fitted curve in the *y*-direction; thus, it is more suitable for predicting the value of one variable by another variable. However, due to the measurement errors in both *x* and *y*, it is not appropriate to only minimize the sum of squared deviations in the *y* direction. Unlike in linear regression models, the standardized major axis estimation determines the minimum distance between the measured value and the fitted curve, and also considers the deviation of the measured value from the fitted curve in both *x* and *y* directions, as well as the slope of the two attributes. Therefore, the standardized major axis estimation is more suitable for the slope estimation of the allometric scaling equation. Standardized major axis estimation was conducted in SMATR^1^, which can test for the heterogeneity of the slopes of different regression equations; *p* < 0.05 indicates that the difference between the slopes is significant, otherwise, a common slope is given. Minitab 17.0 was used to carry out the analysis of variance (ANOVA) and *post hoc* Tukey’s HSD multiple comparison of the stand density and morphological characteristics of *H. ammodendron* and *H. persicum* under different terrain conditions. Origin 8.5 (OriginLab) was used for graphical representation.

## Results and Analyses

### Soil Physical and Chemical Properties of Different Terrains

**FIGURE 2:**
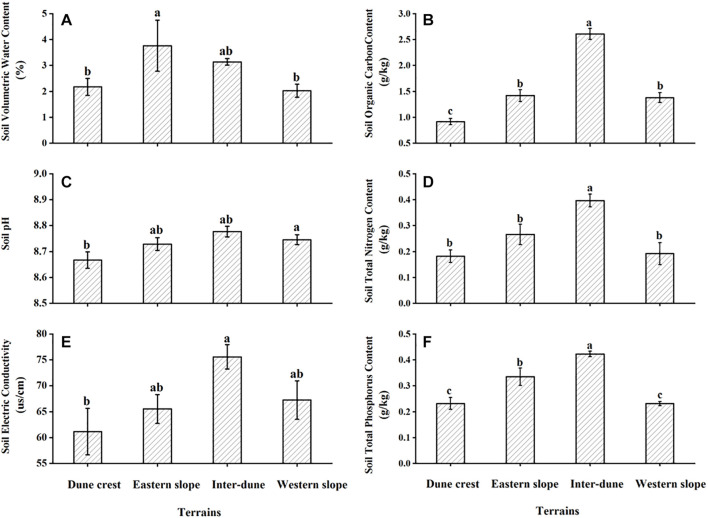
Soil physical **(A)** and chemical properties **(B–F)** of different terrains. Different letters represent significant differences (*p* < 0.05). Data are presented as the mean ± 1 standard error.

Soil physical and chemical properties of different terrains are shown in Figure 2. The results of ANOVA showed significant differences in soil volumetric water content, pH, electrical conductivity, organic carbon content, total nitrogen and total phosphorus content among different terrains (*F* = 3.06, *p* < 0.05; *F* = 3.80, *p* < 0.05; *F* = 3.46, *p* < 0.05; *F* = 72.08, *p* < 0.05; *F* = 12.59, *p* < 0.05; *F* = 21.14, *p* < 0.05). The soil volumetric water content of the eastern slope was the highest; the soil pH, electrical conductivity, organic carbon content, total nitrogen and total phosphorus content of the inter-dune lowland were significantly higher than those of the other terrains (*p* < 0.05). Moreover, the soil volumetric water content of the inter-dune lowland was also relatively high, indicating that the water and nutrient conditions of the inter-dune lowland habitat are superior. On the contrary, soil physical and chemical properties of the dune crest were the lowest.

### Quantitative Characteristics of *H. ammodendron* and *H. persicum* Under Different Terrain Conditions

**FIGURE 3:**
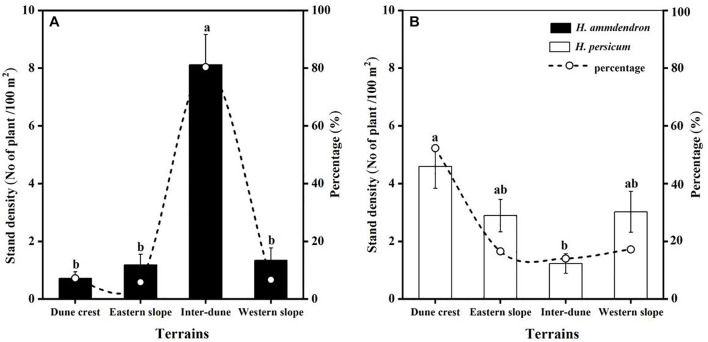
Quantitative characteristic of *Haloxylon ammodendron* **(A)** and *H. persicum* **(B)** at different terrains. Different letters represent significant differences (*p* < 0.05). Data are presented as the mean ± 1 standard error.

**FIGURE 4:**
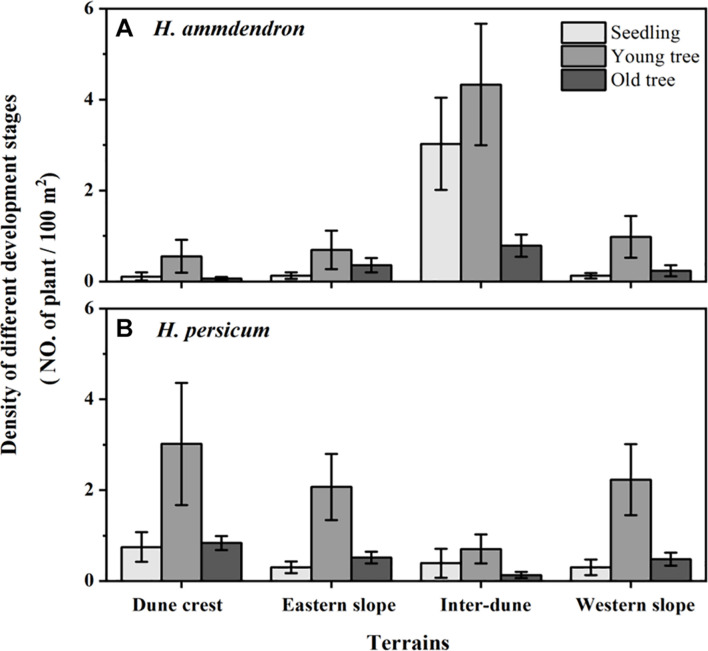
Density of *Haloxylon ammodendron* **(A)** and *H. persicum* **(B)** of different development stage at different terrains. Data are presented as the mean ± 1 standard error.

Among the 336 sampling plots, there were 112 sampling plots located on the dune crest and the inter-dune lowland, respectively, and 56 sampling plots located on the eastern and western slope, respectively. In this study, a total of 2,114 plants were investigated, including 1,131 *H. ammodendron* individuals and 983 *H. persicum* individuals. There were significant differences in stand density of *H. ammodendron* among different terrains (*F* = 27.90, *p* < 0.05), which were in the order of inter-dune lowland > western slope > eastern slope > dune crest. The density of *H. ammodendron* growing in the inter-dune lowland was the highest (8.12 ± 1.04 plants/100 m^2^) (Figure 3), most of them were seedlings and young individuals (Figure 4). Different from *H. ammodendron*, the densities of *H. persicum* were in the order of dune crest > western slope > eastern slope > inter-dune lowland. Only the density difference of *H. persicum* between the dune crest and inter-dune lowland was significant (*p* < 0.05), there was no difference among the other terrains, and the young tree always occupied a larger proportion at each terrain (Figure 4).

### Morphological Characteristics of *H. ammodendron* and *H. persicum* Under Different Terrain Conditions

**FIGURE 5:**
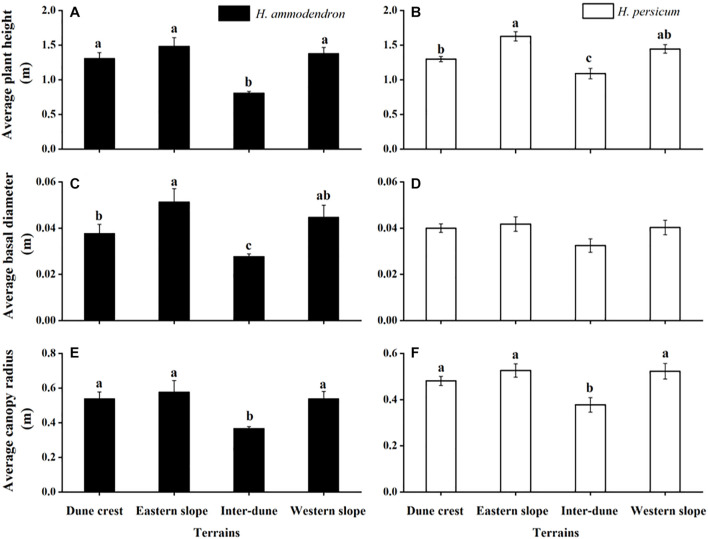
Morphological characteristics of *Haloxylon ammodendron* **(A,C,E)** and *H. persicum* **(B,D,F)** at different terrains. Different letters represent significant differences (*p* < 0.05). Data are presented as the mean ± 1 standard error.

As shown in Figure 5, the patterns of plant morphological characteristics were consistent for *H. ammodendron* and *H. persicum* under different terrain conditions, both were in the order of eastern slope > western slope > dune crest > inter-dune lowland. The plant height, basal diameter, and canopy radius of *H. ammodendron* all showed significant differences among various terrains (*F* = 34.18, *p* < 0.05; *F* = 12.85, *p* < 0.05; *F* = 13.76, *p* < 0.05). The minimum values all appeared in the inter-dune lowland, which were 0.81 ± 0.02, 0.03 ± 0.001, and 0.37 ± 0.01 m, respectively. The plant height and canopy radius of *H. persicum* showed significant differences among various terrains (*F* = 11.40, *p* < 0.05; *F* = 3.89, *p* < 0.05); the maximum values (1.63 ± 0.07 and 0.53 ± 0.03 m) were observed on the eastern slope, while the minimum values (1.09 ± 0.08 and 0.38 ± 0.03 m) were in the inter-dune lowland. The basal diameter exhibited no significant differences (*F* = 1.58, *p* > 0.05).

### Allometric Relationship Between Plant Height and Canopy Radius

Supplementary Figure 1 and Table 2 showed the scatter plot and the quantitative relationship of logarithm-transformed plant height (H) and canopy radius (R) of *H. ammodendron* and *H. persicum*. The log(H)∼log(R) linear regression analysis resulted in the coefficient of determination (R^2^) was greater than 0.7 (*p* < 0.05), indicating a significant positive correlation between H and R of *H. ammodendron* and *H. persicum* under different terrain conditions. Isometric tests showed that only the relationship between the H and R of *H. ammodendron* on the dune crest and inter-dune lowland, and *H. persicum* in the inter-dune lowland were isometric (*F* = 3.64, *p* = 0.06; *F* = 5.05, *p* = 0.08; *F* = 0.01, *p* = 0.93), the relationships among other terrains were allometric (*p* < 0.05). In addition, there was a significant difference in the allometric index (namely the slope) between H and R for *H. ammodendron* and *H. persicum* among various terrain conditions (*F* = 16.07, *p* < 0.05; *F* = 19.23, *p* < 0.05). The allometric indices between H and R of *H. ammodendron* were 1.10, 1.21, 1.03, and 1.20 on the dune crest, eastern slope, inter-dune lowland, and western slope, respectively; while those of *H. persicum* were 1.22, 1.15, 1.00, and 1.15, respectively. The smallest allometric indices between H and R for *H. ammodendron* and *H. persicum* (1.03 and 1.00) both appeared in the inter-dune lowland, and there was no significant difference among other terrains (*p* > 0.05).

**TABLE 2:** Results for SMA fitted slopes between plant height and canopy radius of *Haloxylon ammodendron* and *H. persicum* at different terrains.

Plant Height vs. Canopy Radius	Slopes (95% confidence intervals) *F*, *p* [H_0_: slope (a) = 1]		Intercepts (95% confidence intervals)	
	*H. ammodendron*	*H. persicum*	*H. ammodendron*	*H. persicum*
Dune crest	1.10^ab^ (1.00, 1.22) 3.64, 0.06	1.22^a^ (1.17, 1.26) 105.49, *p* < 0.05	−0.41 (−0.44, −0.37)	−0.50 (−0.51, −0.48)
Eastern slope	1.21^a^ (1.09, 1.34) 14.34, *p* < 0.05	1.15^a^ (1.06, 1.24) 11.57, *p* < 0.05	−0.49 (−0.53, −0.44)	−0.54 (−0.57, −0.51)
Inter-dune	1.03^b^ (1.00, 1.06) 5.05, 0.08	1.00^b^ (0.93, 1.08) 0.01, 0.93	−0.35 (−0.36, −0.34)	−0.48 (−0.51, −0.45)
Western slope	1.20^a^ (1.07, 1.35) 10.46, *p* < 0.05	1.15^a^ (1.07, 1.24) 14.55, *p* < 0.05	−0.45 (−0.49, −0.41)	−0.50 (−0.52, −0.47)
Test statistic, *df, p* H_0_:slope are equal	16.07, 3, *p* < 0.05	19.23, 3, *p* < 0.05		

*Testing for isometric [H_0_: slope (a) = 1, *p* > 0.05] and common slopes (where slopes are equal, *p* > 0.05). Different superscript letters represent significant differences (*p* < 0.05).*

### Allometric Relationship Between Plant Height and Basal Diameter

Similar to the relationship between H and R, the log(H)∼log(D) linear regression analysis indicated a significant positive correlation between the H and D of *H. ammodendron* and *H. persicum* under various terrain conditions (*p* < 0.05). Isometric tests showed that the relationship between H and D of *H. ammodendron* and *H. persicum* under various terrain conditions was allometric (*p* < 0.05), and the allometric indices were significantly different (*F* = 12.15, *p* < 0.05; *F* = 15.01, *p* < 0.05). The allometric indices between H and D of *H. ammodendron* growing on the dune crest, eastern slope, inter-dune lowland, and western slope were 1.30, 1.12, 1.14, and 1.39, respectively, whereas those of *H. persicum* were 1.30, 1.38, 1.15, and 1.15, respectively. The largest allometric indices between H and D for *H. ammodendron* and *H. persicum* (1.39 and 1.38) appeared in the western and eastern slope, respectively.

## Discussion

### Differences in the Distribution of *H. ammodendron* and *H. persicum* Among Various Terrains

In the current study, we found that *H. ammodendron* and *H. persicum* were distributed at all terrains, but the dominant distribution areas of the two species were different. Therefore, the statement “*Haloxylon persicum grows on sand dunes while H. ammodendron* grows on inter-dune lowlands” is inaccurate (Xu et al., 2014). As shown in Figure 3, *H. ammodendron* was concentrated in the inter-dune lowland, accounting for more than 80% of the total, with a density of 8.12 ± 1.04 plants/100 m^2^ (Figure 3). However, *H. persicum* was least distributed in the inter-dune lowland and mostly distributed on the dune crest (accounting for about 50% of the total), with a density of 4.59 ± 0.76 plants/100 m^2^ (Figure 3). The stand density of *H. persicum* on the eastern and western slopes were similar, which were both greater than those of *H. ammodendron*. These results indicate that the distribution patterns of *H. ammodendron* and *H. persicum* are significantly different, the distribution of *H. persicum* is more scattered comparing to *H. ammodendron*.

The difference in the distribution patterns of *H. ammodendron* and *H. persicum* among various terrains is closely related to soil physical and chemical properties of their habitats. Soil is the growth substrate for terrestrial plants, and it is the results of climate, biology, parent material, topography, and time, and is also profoundly affected by modern anthropogenic activities (Weil and Brady, 2016). In desert region, aeolian transport of soil particles greatly affects the spatial distribution of resources, which in turn, exerts significant influence on the growth and recruitment of desert plants (Alvarez et al., 2012; Niu et al., 2021). Studies have shown that the inter-dune lowland and the middle-lower part of the dune in the Gurbantonggut Desert are mainly composed of fixed aeolian sandy soil. In contrast, the middle-upper part and the crest of the dune are mainly composed of semi-fixed aeolian sandy soil (Wang et al., 2003; Zhu et al., 2021). The soil pH, electrical conductivity, water content, and nutrient content of the inter-dune lowland were significantly higher than those of dune crest, as shown in Figure 2, which was in line with other studies (Li C. J. et al., 2011; Xu et al., 2014). To better adapt to the significantly different soil conditions, *H. ammodendron* and *H. persicum* developed different adaptabilities. As proved by habitat soil substrate exchange experiments, *H. persicum* had stronger adaptability and settlement ability on the nutrient-poor dune crest due to its excellent resistance to salt and alkalinity, whereas *H. ammodendron* was more suitable for the inter-dune lowland with a higher soil salt content (Li H. et al., 2011). In addition, sand burial caused by aeolian soil is another important factor controlling the distribution and establishment of desert plant communities (Jia et al., 2011). Previous studies have showed that the ability of *H. ammodendron* to resist wind erosion was weak, but the ability of *H. persicum* to tolerate sand burial and grow in sandy environments was stronger (Hu, 1984; Li H. et al., 2011). Therefore, the differences in soil characteristic of micro-habitats among various terrains and the consequent differences in biological adaptability of the plants together determine the general distribution patterns of *H. ammodendron* and *H. persicum* (Li H. et al., 2011; Xu et al., 2014), and the current study demonstrated that their difference in distribution is not absolute (Figure 3).

### Differences in Morphological Characteristics of *H. ammodendron* and *H. persicum* Among Various Terrains

As previously demonstrated, soil physical and chemical properties determine the distribution patterns of plants, meanwhile, they play important roles in the morphogenesis of plants, especially the soil water supply conditions (Li et al., 2005; Niu et al., 2021). This effect is more prominent for desert plants in arid regions where water supply is limited. Figure 5 showed that the plant height, canopy radius, and basal diameter of *H. ammodendron* and *H. persicum* in the inter-dune lowland were all the smallest, while those on the eastern slope were the largest. This phenomenon may result from the special soil characteristics of inter-dune lowland, including poorly sorted soil sediments, well-developed herb layers which consisting of a large number of ephemeral, ephemeroid and annual plants (as shown in Figure 1B), as well as diverse biological crusts covering the soil which could form an effective permeability barrier: the above factors finally resulted in higher water content in the shallow soil layers (Wang et al., 2003). The superior water and nutrient supplies of the shallow soil layers of the inter-dune lowland (Figure 2) provided suitable conditions for the seed germination of *H. ammodendron*, and triggered a boom of new seedlings (Figures 3, 4). However, due to the limited growth time of newly emerged seedlings, these individuals generally owned smaller height, canopy radius, and basal diameter (Figure 5). On account of the large quantity of new seedling and young tree (Figure 4), the individual morphology of *H. ammodendron* in the inter-dune lowland exhibited a relatively lower stature. On the other hand, the root depth of new seedling and young tree is limited, mainly concentrating at the range of 0–100 cm soil layer (Dan et al., 2007; Huang and Guo, 2009), therefore they principally depended on shallow soil water to survive (Li et al., 2020). Although the overall water status of the inter-dune habitat was relatively better, the water resources competition was fierce considering the large number of *H. ammodendron* seedlings and other desert plants. Under this circumstance, water resources became precious and scarce, which could greatly restricting the survival and development of *H. ammodendron* population in subsequent stages. As the drought intensified, most of the *H. ammodendron* seedlings died (personal observations). Different from *H. ammodendron*, the small individual morphology of *H. persicum* exhibited in the inter-dune lowland may be related to its poor adaptability to high salinity, which limiting its growth and development in this habitat (Lu and Ming, 2012).

The eastern slope of the study area is steep and leeward, with short sunshine time and smaller evaporation, thus the soil water condition is better than the other terrains except the inter-dune lowland (Figure 2). During the field survey, we found relatively few *H. ammodendron* and *H. persicum* seedlings appearing on the eastern slope, instead, there were more big trees existing (Figure 4). These big individuals were usually the ones that surviving from adversities, such as drought and cold, they have developed huge and deep root systems to cope with environmental stresses. According to previous study, the root depth of *H. ammodendron* and *H. persicum* could be up to 13.5 and 18 m, respectively (Sheng et al., 2004; Xu et al., 2017). This allowed them to absorb water from deep soil layers for survival and to be more adaptive to environmental changes (Wu et al., 2019a,b). Therefore, they are advantaged in plant height, canopy radius, and basal diameter (Figure 5). In turn, these advantages help them to take up more light, water and nutrients for life activities, and to occupy an advantageous niche in the competition.

### Allometric Relationship Between H and D, H and R of *H. ammodendron* and *H. persicum*

The morphogenesis of desert trees and shrubs, namely the horizontal expansion (canopy and stem) and vertical growth (height), highlights the coordinative relationship between individual plant growth and environment (Archibald and Bond, 2003; Li S. et al., 2011). In this study, the canopy radius was used to represent the canopy size, so that the characterizing of the allometric relationship was more direct and clear. The results showed that both *H. ammodendron* and *H. persicum* preferred to adopt allometric patterns under various terrain conditions, as the allometric indices between H and R, and H and D were all greater than 1 (Tables 2, 3), which suggested that the relative growth rate of the canopy radius and basal diameter both surpassed that of plant height. Previous drought treatment study has shown that between above- and below-ground, *H. ammdendron* preferentially allocated biomass to the below-ground part (namely roots) along a fixed allometric trajectory, which could benefit the survival and the post-drought recovery of the plants (Zhang et al., 2016). The current study further revealed that *H. ammodendron* also adopted a special allometric strategy for above-ground parts: to allocate more resources to horizontal expansion rather than vertical growth.

**TABLE 3:** Results for SMA fitted slopes between plant height and basal diameter of *Haloxylon ammodendron* and *H. persicum* at different terrains.

Plant height vs. Basal diameter	Slopes (95% confidence intervals) *F*, *p*[H_0_:slope (a) = 1]		Intercepts (95% confidence intervals)	
	*H. ammodendron*	*H. persicum*	*H. ammodendron*	*H. persicum*
Dune crest	1.30^ab^ (1.17, 1.45) 22.91, *p* < 0.05	1.30^a^ (1.24, 1.35) 142.31, *p* < 0.05	−1.61 (−1.65, −1.57)	−1.61 (−1.62, −1.58)
Eastern slope	1.12^bc^ (0.98, 1.27) 3.04, *p* < 0.05	1.38^a^ (1.26, 1.51) 51.16, *p* < 0.05	−1.52 (−1.57, −1.47)	−1.73 (−1.77, −1.69)
Inter-dune	1.14^c^ (1.11, 1.18) 67.13, *p* < 0.05	1.15^b^ (1.06, 1.25) 10.71, *p* < 0.05	−1.50 (−1.52, −1.48)	−1.57 (−1.60, −1.53)
Western slope	1.39^a^ (1.21, 1.59) 24.52, *p* < 0.05	1.15^b^ (1.06, 1.25) 11.59, *p* < 0.05	−1.60 (−1.65, −1.55)	−1.63 (−1.66, −1.60)
Test statistic, *df, p* H_0_:slope are equal	12.15, 3, *p* < 0.05	15.01, 3, *p* < 0.05		

*Testing for isometric [H_0_: slope (a) = 1, *p* > 0.05] and common slopes (where slopes are equal, *p* > 0.05). Different superscript letters represent significant differences (*p* < 0.05).*

The allometric relationship between H and R of studied species might resulted from the increased xylem water transport resistance: as the plant height increases would result in the decrease of water transported to the top of plant. In response, plants lowered the stomatal conductance through the physiological adjustment to reduce water loss, meanwhile, the absorption of CO_2_ as the raw material for photosynthesis was inevitably affected. This led to a decreased ability of plants to assimilate carbon, which ultimately restricted the heights of plants (Shi et al., 2015). This has been confirmed in the study of Zhang et al. (2009), in which they showed that the number of dead branches and the individual mortality rate increased as the height of a tree increased in a savanna, because along with this process, their water transport system and water physiology developed into the direction that were more prone to water shortage. In addition, the study area is a typical desert ecosystem with abundant light resource and sparse plant cover, the above-ground parts of vegetation distribute intermittently, thus unlike some dense forest ecosystems, light is not an important limiting factor in the intraspecific and interspecific competition (Li S. et al., 2011). This might be the other reason why the growth of plant height became slow: they don’t need to grow higher to get light. Similarly, the trade-off allocation of resources reflected in the allometric relationship between H and D of studied species was also an indicator of plant response to numerous selection pressures and constraints. Therefore, the allometric relationships between H and R, H and D could be regarded as an important feature of plants adaptability to heterogeneous environments, which enables them to optimally allocate various resources to different parts and achieve maximum benefit for survival and growth (Jacob, 2004; Nicotra et al., 2010; Hou et al., 2014; Turcotte and Levine, 2016).

Within a limited spatial scale, topography indirectly affects plant growth dynamics and species distribution through redistribution of soil water, nutrients and light resources (Zhao et al., 2019; Tang et al., 2020). As shown in Tables 2, 3, the allometric indices between H and R, H and D of two *Haloxylon* species were significantly different among various terrains, this might own to the habitat heterogeneity at different terrains, and also might be related to the ecological adaptability of two studied species in heterogeneous habitats. In the study area, western wind prevails all year around, the wind speed gradually increases from the bottom of the dune along the slope, and reaches the maximum on the dune crest (Wang et al., 2003). The eastern, western slope and the dune crest habitats have a windy environment with complicated water conditions (Figure 2). The allometric indices between H and R of studied species on these three terrains were greater than that of inter-dune lowland (Table 2), suggesting that *H. ammodendron* and *H. persicum* preferentially invested more photosynthetic products into the canopy growth instead of plant height. This might because taller plants face unnecessary pressures in a windy environment, like being broken off. However, in the inter-dune lowland with less wind, most of individuals were seedlings that did not reached their growth limit (Figure 4), they adopted an isometric allocating mode between H and R (Table 2). As an important link of above- and below-ground part, the basal stem of plant connects the trunk upward and the root downward. Although the overall allometric relationship between H and D confirmed that studied species preferentially meet the requirements of water and nutrient transport as well as mechanical support (Brüchert and Gardiner, 2006; Shi et al., 2015), the terrain had a significant effect on it, and the effect was quite different between these two species. According to earlier studies, the basal diameter would increase when plants were mechanically stimulated (Goodman and Ennos, 1996), and stems with a larger basal diameter could transport water under a low tension of xylem vessels (Zhao et al., 2013). As shown in Table 3, the largest allometric index between H and D (1.39) for *H. ammodendron* appeared in western slope, where was windward and water-limited (Figure 2). It suggested that *H. ammodendron* was inclined to invest more into the basal diameter growth, so as to resist the mechanical stimulation caused by long-term unidirectional wind and to maintain a high water transport capacity. In contrast, *H. persicum* growing on the eastern slope, where the water supply is relatively sufficient (Figure 2), promoted investment into its basal diameter, as confirmed by the largest allometric index between H and D (1.38). A larger basal diameter corresponded to a bigger cross-sectional area of the xylem, meaning a higher water and nutrient transport capacity, large stem could help plants to access to resources efficiently. The differences in the allometric relationship between H and R, H and D of *H. ammodendron* and *H. persicum* among various terrain conditions indicated their ecological adaptability to heterogeneous environments, and also revealed the diversification of plants to adapt to various environments.

## Conclusion

In the current study, through investigating two dominant species in the Gurbantunggut Desert of Central Asia, we found that the above-ground parts of *Haloxylon ammodendron* and *H. persicum* both adopted a fixed allometric pattern, as they preferentially invested into plant canopy radius and basal stem instead of plant height. This indicated that the above-ground parts of desert plants were more in favor of horizontal expansion rather than vertical growth, which might be closely related to the environmental characteristics of resource-deficient but light-rich. The stand densities and individual morphology of the two *Haloxylon* species exhibited distinct differences among different terrains, suggesting that the topography could significantly affect the plant growth dynamics. In addition, the allometric indices between H and R, H and D of studied species also significantly varied among terrains although they along a fixed allometric trajectory, indicating that they could finely control the investment in main functional traits in changing environment. These findings contributed to the understanding of the adaptation strategies employed by desert plants, and how changes in terrains within in a limited spatial scale influenced them.

## Data Availability Statement

The raw data supporting the conclusions of this article will be made available by the authors, without undue reservation.

## Author Contributions

YL conceived the study and critically reviewed and edited the manuscript. XW, X-JZ, and X-HM conducted the experiments. XW analyzed the data and drafted the manuscript. All authors have contributed to the study conceptualization and read and approved the final version of this manuscript.

## Conflict of Interest

The authors declare that the research was conducted in the absence of any commercial or financial relationships that could be construed as a potential conflict of interest. The reviewers DZ and LT declared a shared affiliation, with no collaboration, with several of the authors X-JZ and X-HM to the handling editor at the time of the review.

## Publisher’s Note

All claims expressed in this article are solely those of the authors and do not necessarily represent those of their affiliated organizations, or those of the publisher, the editors and the reviewers. Any product that may be evaluated in this article, or claim that may be made by its manufacturer, is not guaranteed or endorsed by the publisher.

## Footnotes

1
http://bio.mq.edu.au/ecology/SMATR
