# Supplementary material for: Differences in Allometric Relationship of Two Dominant Woody Species Among Various Terrains in a Desert Region of Central Asia
Source: Front Plant Sci. 2021 Nov 10;12:754887. doi: 10.3389/fpls.2021.754887 (PMC8631541; doi:10.3389/fpls.2021.754887)
Supplement: Supplementary file 1 [file Data_Sheet_1.docx]

**Supplementary material**

**Differences in allometric relationship of two dominant woody species among various terrains in a desert region of Central Asia**

Wu Xue^1,2,3^, Zheng Xin-Jun^4,5^, Mu Xiao-Han^4,5^, Li Yan^6,*^

^1^College of Resources and Environment Science, Xinjiang University, Urumqi 830046, China

^2^Key Laboratory of Oasis Ecology (Ministry of Education), Xinjiang University, Urumqi 830046, China

^3^Ecological Postdoctoral Research Station, Xinjiang University, Urumqi 830046, China

^4^State Key Laboratory of Desert and Oasis Ecology, Xinjiang Institute of Ecology and Geography, Chinese Academy of Sciences, Urumqi 830011, China

^5^Fukang Station of Desert Ecology, Chinese Academy of Sciences, Urumqi, Fukang 831505, China

^6^State Key Laboratory of Subtropical Silviculture, Zhejiang A&F University, Lin’an 311300, China

*** Correspondence:**Corresponding Author
liyan2016@zafu.edu.cn

| 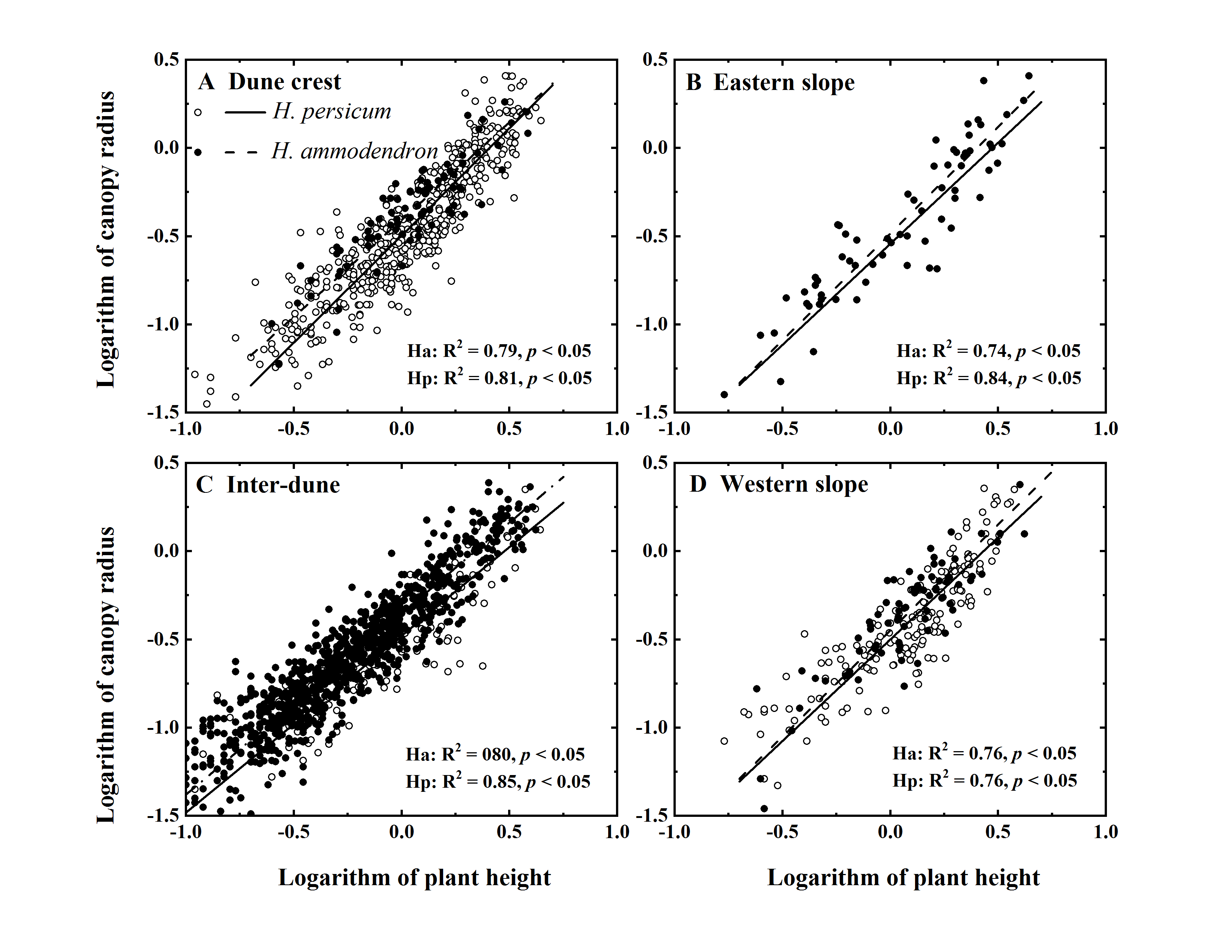 |
| --- |
| Figure S1 Allometric relationships between plant height and canopy radius of *Haloxylon ammodendron* and *H. persicum* at different terrains |
| 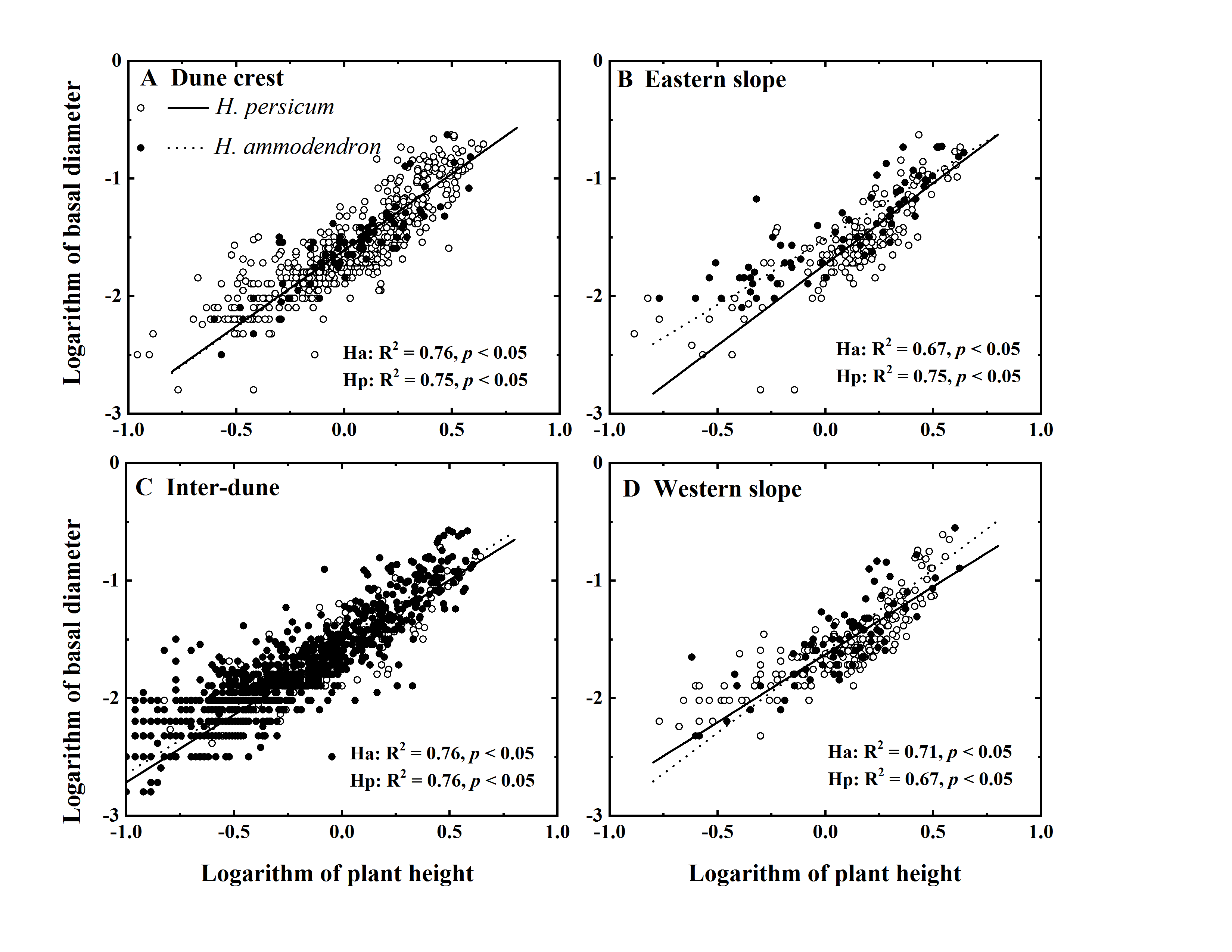 |
| Figure S2 Allometric relationships between plant height and basal diameter of *Haloxylon ammodendron* and *H. persicum* at different terrains |
